# Multi-Probe Measurement System Based on Single-Cut Transformation for Fast Testing of Linear Arrays

**DOI:** 10.3390/s21051744

**Published:** 2021-03-03

**Authors:** Fernando Rodríguez Varela, Manuel José López Morales, Rubén Tena Sánchez, Alfonso Tomás Muriel Barrado, Elena de la Fuente González, Guillermo Posada Quijano, Carlos Zarzuelo Torres, Manuel Sierra Pérez, Manuel Sierra Castañer

**Affiliations:** 1 Radiation Group, Universidad Politécnica de Madrid, 28040 Madrid, Spain; f.rodriguezv@upm.es (F.R.V.); manuelop@ing.uc3m.es (M.J.L.M.); ruben.tena-sanchez@mvg-world.com (R.T.S.); at.muriel@upm.es (A.T.M.B.); e.delafuenteg@alumnos.upm.es (E.d.l.F.G.); manuel.sierra.perez@upm.es (M.S.P.); 2 Indra Sistemas S.A., 28830 Torrejón de Ardoz, Spain; gposada@indra.es (G.P.Q.); czarzuelo@indra.es (C.Z.T.)

**Keywords:** antenna measurements, multi-probe, single-cut transformation, linear array, plane wave spectrum, anechoic chamber

## Abstract

This paper introduces a near-field measurement system concept for the fast testing of linear arrays suited for mass production scenarios where a high number of nominally identical antennas needs to be measured. The proposed system can compute the radiation pattern, directivity and gain on the array plane, as well as the array complex feeding coefficients in a matter of seconds. The concept is based on a multi-probe antenna array arranged in a line which measures the near field of the antenna under test in its array plane. This linear measurement is postprocessed with state-of-the-art single-cut transformation techniques. To compensate the lack of full 3D information, a previous complete characterization of a “Gold Antenna” is performed. This antenna is nominally identical to the many ones that will be measured with the proposed system. Therefore, the data extracted from this full characterization can be used to complement the postprocessing steps of the single-cut measurements. An X-band 16-probe demonstrator of the proposed system is implemented and introduced in this paper, explaining all the details of its architecture and operation steps. Finally, some measurement results are given to compare the developed demonstrator with traditional anechoic measurements, and show the potential capabilities of the proposed concept to perform fast and reliable measurements.

## 1. Introduction

Far-Field (FF) radiation pattern is one of the most important parameters to evaluate antenna performance. Moreover, other important validation parameters, such as complex feeding coefficients in the case of arrays, may be extracted from it. Near-Field (NF) measurement ranges [1,2,3] constitute a versatile and accurate antenna characterization tool. By measuring the Antenna Under Test (AUT) radiated field over planar [4], cylindrical [5] or spherical [6] surfaces, the FF pattern can be derived more efficiently than with traditional FF techniques. Antenna measurement processes tend to be time-consuming, so there is a continuous need of improving the existing techniques and developing new ones to maximize the efficiency and promptness. One clear example is the testing of antennas produced in serial manufacturing, i.e., base station antennas for 5G communications, active antennas for satellite tracking or radar antennas, which are based on linear or planar arrays and where the pattern measurement is usually the bottleneck of the process.

Multi-Probe Measurement Systems (MPMS) [1] offer an alternative method to drastically downsize the measurement time. Instead of moving a probe over a certain surface to perform the measurement, an array of probes is arranged to sample specific points of this surface. An example may be found in [7], where a full NF spherical measurement is acquired by a MPMS settled along a ring which only rotates on its axis. The same principle may be applied to other scanning surfaces resulting in a promising alternative to achieve fast antenna measurements.

Some applications do not require the full 3D characterization of the AUT radiation, but one or two of the FF radiation pattern principal planes (and its associated gain or directivity). Nevertheless, it is necessary to perform measurements over the complete scanning surface to perform the classical NF to FF transformations [1]. This may lead to excessive measurement times when a high number of AUT’s must be verified. Furthermore, this problem becomes even worse when validating arrays with beam-steering capabilities so more than one FF radiation pattern is acquired per AUT. In this context, it is convenient to lose some accuracy in exchange of a measurement time reduction by performing a one-by-one Single-Cut Transformation (SCT) [8,9,10].

Some efforts have been made to develop fast antenna characterization techniques based on this SCT concept. In [8] spherical and cylindrical wave expansions are used to perform NF to FF transformations over rings enclosing the AUT, with good agreement with full spherical measurements. An equivalent currents approach is proposed in [9] to predict the full FF radiation pattern of long linear arrays. Applying some approximations, the FF is predicted only by measuring the AUT in a line parallel to the array and a perpendicular ring enclosing it, both in the reactive-field region. The authors in [10] propose to perform SCT’s over arbitrary curves expanding the antenna radiated fields into propagating plane waves by means of a 2-D particularization of the Fast Multipole Method.

The aforementioned SCT’s rely on the separability of the antenna radiated fields, which consists of assuming that the 3-D antenna radiation pattern in U,V coordinates ($E\left(U,V\right)$) can be expressed as the product of the radiation patterns of the two principal planes [11]:

(1) $$E\left(U,V\right)=E_{U}\left(U,V=0\right)\;\cdot \;E_{V}\left(U=0,V\right)$$

where $E_{U}\left(U,V=0\right)$ and $E_{V}\left(U=0,V\right)$ are the horizontal and vertical cuts respectively, for any of the electric field components radiated by the AUT. Even though this condition does not hold for a general case, it is met for some types of antennas. For planar arrays with separable complex feeding excitation coefficients, Equation (1) can be derived straightforwardly thanks to the separability of the exponentials involved in the array factor computation [12]. In the case of linear arrays, one of the planes is dominated by the individual element radiation pattern and the other by the array factor. Hence, if the array is arranged in the $\hat{x}$ or $\hat{y}$ axis, Equation (1) becomes a good approximation of the radiation pattern. In the case of planar arrays, the full 3-D radiation pattern is mainly influenced by the array factor, which is usually designed to be separable. However, manufacturing errors affecting either the individual elements or the feeding network in both dimensions break the separability condition, which may lead to a performance degradation of the applied SCT techniques.

This paper presents an antenna measurement system which combines the versatility of MPMS’s with the principles of SCT NF to FF transformation for measurement time enhancement. From now on we will refer to this system as SCT-MPMS (Single-Cut Transformation Multi-Probe Measurement System). The SCT-MPMS consists of a set of linearly polarized probes arranged in a line in front of the AUT. A switching matrix is connected to the probe array to perform the complete measurement process virtually at once. This set-up allows to retrieve the FF radiation pattern of linear arrays on the array cut, to evaluate its gain and directivity as well as to estimate the array complex feeding coefficients. By comparing the obtained coefficients with the theoretical ones, it is possible to detect a particular amplitude or phase imbalance in the feeding network or an element poorly fabricated. Thus, any fabrication error may be not only detected, but also located in the malfunctioning element/s, and its impact on the overall behavior of the AUT can be assessed.

For an appropriate operation of the proposed SCT-MPMS, a previous calibration procedure is mandatory. This calibration step requires a full traditional measurement of a gold standard antenna, which is just a working antenna selected from the many ones that will be measured in series with the SCT-MPMS. After that, the AUTs of the same antenna model can be measured extremely fast. Therefore, this system is suited to mass production scenarios where a high number of nominally identical antennas needs to be measured. This concept may be extended to any polarization and AUTs by adapting the SCT-MPMS and its postprocessing steps. In case the AUT is an electrically steered antenna, a phase shifting control could be included in the procedure, so the measurement of every beam-steered position is coordinated and automatized in the same iteration. This paper will show a demonstrator which does this integration resulting in an additional measurement time enhancement. This demonstrator constitutes only a proof-of-concept of the SCT-MPMS, whose performance has been tested in an anechoic environment. At the very last step, the entire system could be integrated in an ad-hoc anechoic box that allows to place one AUT after another, automatizing the rest of the process.

The remainder of this paper is as follows. Section 2 presents a description of the proposed SCT-MPMS. Section 3 depicts the calibration measurement step, Section 4 analyzes the measurement and postprocessing techniques performed, and Section 5 shows some empirical results using linear phased arrays as AUT. After a discussion on the potentials and limitations of the proposed measurement system in Section 6, the paper is concluded in Section 7.

## 2. Single-Cut Transformation Multi-Probe Measurement System Architecture

**Figure 1:**
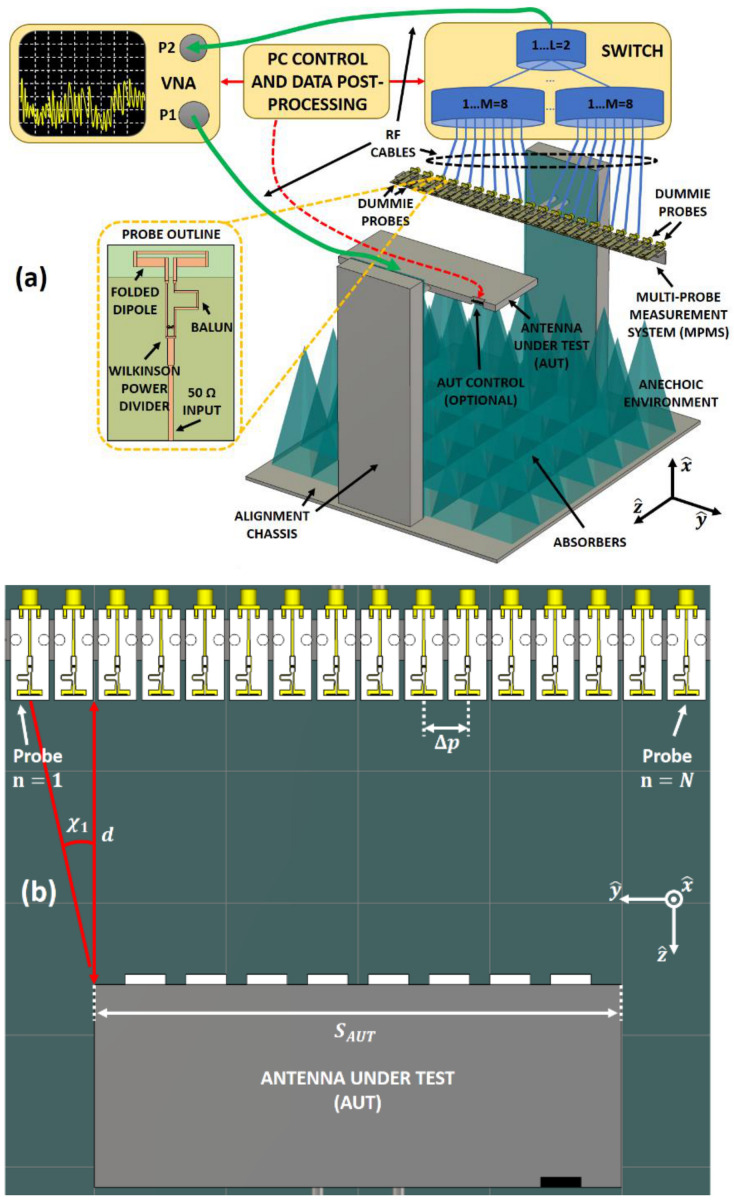
Proposed Single Cut Transformation Multi-Probe Measurement System (SCT-MPMS). (**a**) General scheme of the SCT-MPMS. (**b**) Top view of the SCT-MPMS probe array vs. Antenna Under Test (AUT) and its reliable angle.

As previously mentioned, the purpose of the proposed SCT-MPMS is to validate linear array antennas in a very fast and as accurate way compared to traditional techniques. Therefore, applications where many linear arrays need to be tested take advantage of a considerable time saving. The general scheme behind the proposed SCT-MPMS is depicted in Figure 1a. It is composed of a certain number *N* of probes arranged in a linear fashion in front of the NF area of the AUT. All the probes are connected to a switching matrix that commutes between them to sample different NF points of the AUT in the same iteration. This matrix may be multi-leveled depending on the number of probes. Besides this, a Vector Network Analyzer (VNA) is connected to the AUT input and the switching matrix output to perform the frequency sweep and measure the S_21_ parameter. Consequently, the switching matrix and the VNA control are properly coordinated by a computer so every probe samples a point in each measurement iteration. Then, all the acquired samples are stored and post-processed in the same computer in order to evaluate the desired parameters. The proposed system is designed for a generic linear array, which may include a feeding amplitude or phase control. If the AUT is an electronically steered array, its control interface is integrated in the measurement routine. Thus, the measurement of all the patterns that the AUT can generate is performed in the same iteration, leading to an additional time saving.

Figure 1b shows a top view of the scheme of the proposed SCT-MPMS. As it can be seen, the probes are settled with a separation between probes of Δ*p* and a distance *d* between the probes and the AUT. Both the AUT and probes need to be perfectly centered and aligned one with respect to the other. Like in a planar measurement system, a truncation on the measured radiation pattern up to an angular value of $\chi_{1}$ arises [3]. As it can be appreciated in Figure 1b, this angle will depend on the probe to AUT distance *d*, the size of the AUT in the measurement plane S_AUT_, the number of probes *N*, and their separation Δ*p*. Apart from the inherent truncation of planar measurement systems, the proposed SCT-MPMS suffers from other kind of limitations that the postprocessing must deal with:

1. Increasing the number of probes may improve the reliable angular margin $\chi_{1}$ and enhance the postprocessing with a higher number of samples to compute. This leads to lower truncation error, but it increases the switching matrix complexity.
2. In principle. the separation between probes Δ*p* must be below $\frac{\lambda }{2}$ in order to meet the Nyquist criterion for planar measurement systems (explained in Section 4). However, this requirement may be relaxed so this error does not affect the reconstructed radiation pattern in the reliable angular margin.
3. Any alignment error due to mechanical imperfections will lead to an invalidation of the separability condition.

**Figure 2:**
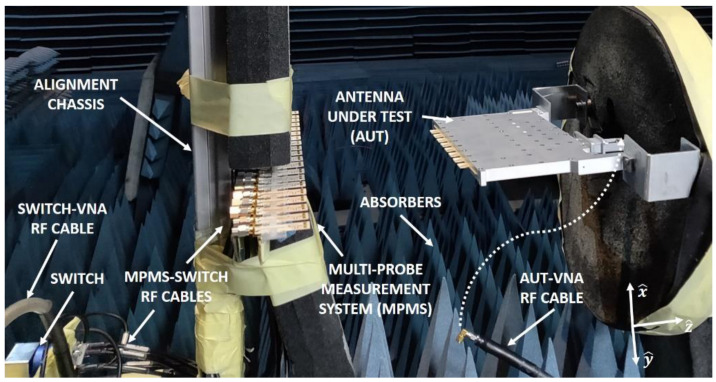
Demonstrator prototype of the proposed SCT-MPMS inside an anechoic chamber.

The presented system is extensible not only to other type of AUT’s, but also to other frequency bands and different polarizations by adapting the probe design and by taking into consideration the different limitations of these systems. Figure 2 shows the demonstrator of the presented SCT-MPMS, that operates at X-band. It consists of an array of *N* = 16 probes connected to a switching matrix split into two different division levels of *L* = 2 and *M* = 8. The AUT is perfectly aligned with the probe array at a distance of *d* = 18 mm as a compromise between AUT-probe coupling and truncation angle $\chi_{1}$. The antennas designed as probes are printed folded dipoles fed by a combination of a Wilkinson power divider and a balun (see Figure 1a). The probe separation is fixed to Δ*p* = 21.6 mm to fulfill with the aliasing condition within the reliable angular margin. In addition, this separation leads to an insignificant coupling between probes that are two-element separated. A pair of two dummy probes are arranged besides the first and the last probes of the array so the radiation pattern of each element is as steady as possible independently from the probe. All these dimensions have been selected to perform a NF measurement system at 10 GHz. At this frequency, the AUT to probe distance is *d* = 5λ and the probe separation is Δ*p* = 0.7λ.

As pointed out before, the measurements carried out with this demonstrator were performed in the anechoic chamber. Once the performance of the demonstrator is validated, the entire SCT-MPMS can be integrated inside an ad-hoc anechoic box which includes all the proposed hardware and software.

## 3. SCT-MPMS Measurement Procedure

This section gives a detailed description of how to carry out a particular set of measurements on the SCT-MPMS. As previously mentioned, the proposed system is suited for applications where a high number of nominally identical AUTs need to be verified as rapidly as possible. Therefore, from this section onwards, all the AUTs to be measured and validated are going to be prototypes of the same antenna model. These prototypes are going to be addressed as clone AUTs.

**Figure 3:**
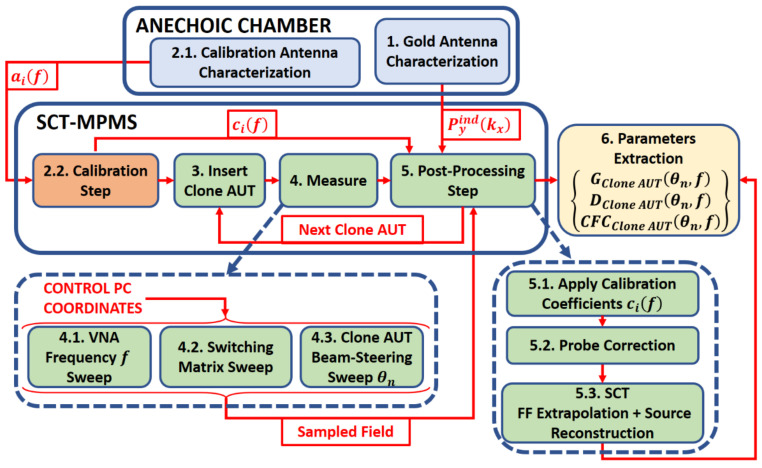
General scheme of the measurement process. The continuous blue rim boxes denote the steps that are performed either in a standard anechoic chamber or in the proposed SCT-MPMS system.

Figure 3 illustrates a general step-by-step scheme of the measurement process. It applies to the proposed demonstrator in this paper and as well as to any other implementation of the SCT-MPMS. Throughout this process, the two different stages that are discussed deeper in this section and in Section 4 are mentioned: the calibration and postprocessing steps. Both sub-routines need previous information from reference antennas to fulfill their goal. A previous characterization of these antennas is then needed. The specific stages in which the measurement process has been divided are:

1. Gold Antenna characterization. For the postprocessing of the measurements, a Gold Antenna is needed to compute the radiation parameters of all the clone-AUT’s to measure. The Gold Antenna also needs to be a clone of the same type. Thus, this antenna is fully characterized in a conventional NF measurement. The Gold antenna is then measured in the SCT-MPMS to extract the radiation information that will be required during the postprocessing steps.
2. Calibration Antenna characterization and calibration step. The calibration step also needs a reference antenna to perform an efficient RF calibration of the SCT-MPMS. This calibration is required to equalize the different channels of the probes. Therefore, a calibration antenna is characterized in order to extract a set of complex coefficients which can be applied to the rest of measurements, making the switch and cables effects negligible. This step is detailed in Section 3.1.
3. Insert clone AUT. The first clone AUT is introduced in the SCT-MPMS an aligned. In the proposed demonstrator, this alignment may be a time-consuming process, but in a final implementation this can be designed with the appropriate mechanical fixtures to enable a fast, automatized and simple alignment process.
4. Measure. The control Personal Computer (PC) performs the frequency *f* sweep on the VNA while the switching matrix changes the measuring probe in a coordinated way. Different beam-steered θ_n_ patterns may be acquired at once since the clone-AUT control can be integrated in the measurement process.
5. Post-processing. The obtained NF samples are weighted by the calibration coefficients to compensate for the cable and matrix path differences. Then, they are processed by the control PC that performs a probe correction, FF extrapolation and source reconstruction on them. Besides, it uses the information from the Gold Antenna to compensate the lack of 3D NF information. These algorithms are detailed in Section 4.
6. Parameter Extraction. The desired parameters from the clone AUT are available so its performance can be validated. The next clone AUT is ready to be introduced in the SCT-MPMS, which may be possible to be done while the postprocessing is being carried out, in order to maximize time efficiency.

It is important to evaluate the time-enhancement of the measurement process: with only two traditional antenna measurements (Calibration and Gold antennas) it is possible to measure as many clone AUTs as needed. Finally, it is noted that the Gold and Calibration antennas may be the same one. The only requirement is that the Gold one is of the same type as the rest of clone AUTs to allow for an accurate postprocessing. 

### 3.1. Calibration Antenna Characterization

**Figure 4:**
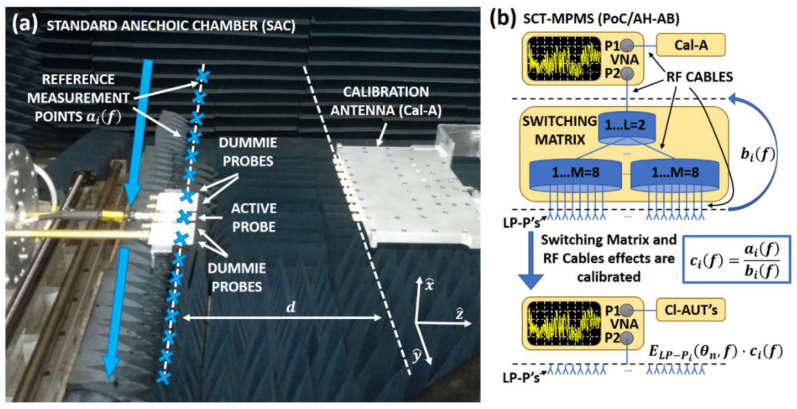
Calibration antenna characterization process in: (**a**) Conventional anechoic chamber planar measurement and (**b**) the SCT-MPMS.

Ideally, all probes should receive the complex value of the AUT radiated field at each point. Nevertheless, the switching matrix may not behave in the same way for all the possible paths, and the cables may not be exactly the same due to imperfections and displacements when the AUT is changed or the SCT-MPMS is moved. These are frequent sources of imbalances between the measurement points, so every time the set-up is altered in any way it is necessary to perform a calibration procedure of the SCT-MPMS. Hence, a Calibration Antenna is selected to be measured in the SCT-MPMS to obtain the calibration coefficients for the specific set-up. The Calibration Antenna may be any kind of antenna that is characterized and used for calibration as follows:

1. Firstly, a planar NF measurement of the Calibration Antenna is performed over a line i.e., single-cut, as it can be seen in Figure 4a. This emulates the measurement performed by the SCT-MPMS but with a single moving probe. So, in the case of our demonstrator, 16 NF samples are recorded at the same virtual locations of the probe array elements. In fact, the probe used for this measurement is a printed dipole with four adjacent dummies identical to the ones of the SCT-MPMS. This is a fair approximation of the real SCT-MPMS since the probe empirically show a coupling factor below -18 dB for the dipoles separated more than two elements. The acquired NF samples from this measurement are the $a_{i}\left(f\right)$ coefficients in Figure 4.
2. Secondly, the Calibration Antenna is measured in the SCT-MPMS obtaining an additional set of NF samples $b_{i}\left(f\right)$ (see Figure 4b). The channel imbalance can be assessed by comparing these samples with the ones of the previous measurement, the different channels imbalance, $a_{i}\left(f\right)$. The ratio between both set of measurements is performed, thus obtaining the calibration coefficients $c_{i}\left(f\right)$ (see Figure 4b upper part). The obtained calibration coefficients can be applied to the subsequent measurement samples of the clone antennas to cancel for the RF cables and the switching matrix effects (see Figure 4b lower part). For a proper calibration, the positioning errors between both set of measurements must be low enough (a misalignment error of 1 mm would lead to a 12° phase error at 10 GHz).

### 3.2. Gold Antenna Characterization

The applied SCT technique assumes that all clone AUTs from a given antenna model have the same horizontal plane pattern. This horizontal cut must be obtained before starting the serial measurements with the SCT-MPMS and this is done thanks to the Gold Antenna. Therefore, the first time that a batch of clone AUTs need to be characterized, a conventional full measurement process of its corresponding Gold Antenna is performed (first stage in Figure 3). The obtained horizontal cut is used in the postprocessing steps of the rest of clone-AUTs (5th stage in Figure 3), as it is assumed to be steady among them.

Thus, a high number of clone-AUTs of the same antenna model can be validated very quickly. The only stage that takes significant time is the Gold and Calibration Antenna characterization, but it is negligible compared to the time saved in characterizing the rest of the clone AUTs. It is noted that in the present paper the Gold and Calibration Antenna will be the same one, allowing a considerable time-enhancement.

## 4. SCT-MPMS Postprocessing Step

This section explains in detail the 5th stage of the presented measurement outline in Section 3 (see Figure 3). It focuses on the postprocessing after calibration and probe correction once the radiated field is sampled by the probe array for every frequency f and pointing direction θ_n_. The presented procedure is then able to extract the desired parameters of interest: gain and directivity in the array plane and the complex feeding coefficients. The sub-steps comprising this process may be divided into two different categories:

1. NF to FF SCT.
2. Parameters extraction.

These two stages are explained in more detail in the following subsections, where the mathematical principles of the postprocessing are discussed.

### 4.1. Near-Field to Far-Field Single-Cut Transformation

As previously stated, after the SCT-MPMS calibration and probe correction, the samples acquired by the probe array need to be post-processed to extract the defined antenna parameters. Since the probes measure the AUT NF only over a line, a FF transformation is required. The NF to FF transformation is performed using the Plane Wave Spectrum (PWS) formulation [4], that fundamentally consists of applying a Fourier Transform over the radiated field of the antenna. However, it needs to be particularized to the case where only information across one dimension is available.

The field radiated by the AUT admits a plane wave expansion in the z > 0 region:

(2) $$\vec{E}(\vec{r})=\frac{1}{2\pi }\iint_{-\infty }^{\infty }\vec{P}\left(k_{x},k_{y}\right)e^{-j\hat{k}\vec{r}}dk_{x}dk_{y}$$

where $\vec{P}\left(k_{x},k_{y}\right)$ is the AUT PWS and $\vec{E}(\vec{r})$ is its radiated field. The proposed demonstrator works with only one polarization, so the vertical $\hat{y}$ polarizarion of the PWS: $P_{y}\left(k_{x},k_{y}\right)$ is going to be used in the rest of derivations. As for the $\hat{z}$ polarization of the PWS, this one is derived enforcing the plane wave condition where the x component is assumed to be 0, which is a reasonable approximation for linear polarized antennas. 

In the case of a linear array arranged on the $\hat{y}$-axis, and assuming separability of the array, the PWS can be denoted as the product of the PWS of the array individual element $P_{y}^{ind}$ and the array factor (AF) [11]:

(3) $$P_{y}\left(k_{x},k_{y}\right)\;\approx P_{y}^{ind}\left(k_{x}\right)\cdot AF\left(k_{y}\right)$$

where the $k_{y}$ dependence of the individual element PWS has been neglected since the main contribution on this plane is given by the array factor. With these considerations, Equation (3) can be particularized in Equation (2) to express the $E_{y}$ field component over a line parallel to the array at a distance $z_{0}$:

(4) $$E_{y}\left(x=0,y,z=z_{0}\right)=\frac{1}{2\pi }\iint_{-\infty }^{\infty }P_{y}^{ind}\left(k_{x}\right)\;AF\left(k_{y}\right)e^{-j(yk_{y}+z_{0}k_{z})}dk_{x}dk_{y}$$

Taking Inverse Fourier Transform at both sides of Equation (4) allows to isolate the array factor $AF\left(k_{y}\right)$. Thus, it is possible to compute the array factor from a single measurement line substituting due to the mentioned PWS separability:

(5) $$AF\left(k_{y}\right)=\frac{\int_{-\infty }^{\infty }E_{y}\left(x=0,y,z=z_{0}\right)e^{jyk_{y}}dy}{\int_{-\infty }^{\infty }P_{y}^{ind}\left(k_{x}\right)e^{-jz_{0}k_{z}}dk_{x}}$$

The numerator of Equation (5) involves the calculation of the Fourier Transform of the measured field in a line. This can be done using the measured samples by the probe array. The denominator, however, requires the knowledge of the AUT individual element in the other plane, which is not measured by the SCT-MPMS. However, we can take the term $P_{y}^{ind}\left(k_{x}\right)$ obtained from the postprocessing of the Gold Antenna previously characterized and use it for the rest of the clone antennas. Of course, this is an approximation but as mentioned before, most of the information and potential failures will be located on the plane of the array.

The integral shown in Equation (5) extends to infinite, but the measured field is only available in a given finite region. This truncation will limit the transformed radiation pattern to a given angular sector called reliable region (from −$\chi_{1}$ ≤  θ ≤ $\chi_{1}$ in Figure 1b). This is a typical problem in planar near-field antenna measurements which is alleviated using an extrapolation technique known as the Gerchberg-Papoulis algorithm [13]. This algorithm is able to reconstruct truncated signals by exploiting the information about its band-limitation properties. The algorithm consists in an iterative process, alternating between the signal and its spectral domains. In the spectral domain, a filtering is applied since the bandwidth of the truncated signal is a priori known, while in the signal domain the resulting truncated part is updated on every iteration. In this application, the truncated signal is the radiation pattern cut in the reliable region while the spectrum is the one-dimensional extent of the AUT aperture, S_AUT_. Therefore, spatial filtering and spectral substitutions are applied iteratively so that the radiation pattern is extrapolated outside of the reliable region. The Gerchberg-Papoulis algorithm has been used previously [14] to reconstruct the far-field pattern outside the reliable region from truncated planar near-field measurements with remarkable results. In this case, such algorithm has been implemented in a similar manner particularized to the one-dimensional case.

Another practical consideration is that the integrals of Equation (5) must be evaluated numerically, so the discrete versions of the spectrums and fields must be used. $P_{y}^{ind}$ can be obtained at the desired sampling rate from the Gold Antenna characterization but $E_{y}$ is only measured at a discrete set of points in the SCT-MPMS. According to the planar-near field transformation theory [3], the minimum separation *Δp* between samples to obtain the radiation pattern in an angular region $\left|\theta \right|<\alpha_{0}$ free of aliasing is:

(6) $$\Delta p=\frac{\lambda }{2\sin\alpha_{0}}$$

Therefore, the separation between probes Δ*p* is set following the criterion in Equation (6) depending on the desired value of $\alpha_{0}$. This value is adjustable depending on the AUT size. In the proposed SCT-MPMS the value of $\alpha_{0}$= 45° is chosen, which leads to the selected separation between probes of Δ*p* = 0.7λ. (see Figure 1b). Furthermore, the proposed SCT-MPMS can measure AUTs of a maximum size of S_AUT_ =18 cm in order to have a minimum reliable region of $\chi_{1}$ = 20°, which coincides with the minimum angular region in which the Gerchberg-Papoulis was found to extrapolate the radiation pattern with good accuracy for AUTs of such size.

Finally, it should be noted that the computed value of $AF\left(k_{y}\right)$ will be influenced by the probe radiation pattern, so a standard probe correction is applied [3]. In order to do this, the probe radiation pattern must be characterized, or a simulation model may be used. In the case of the proposed demonstrator, the probe used for the calibration has been fully characterized and its measured radiation pattern is used to apply the probe correction, assuming that all probes show the same pattern. This is a reasonable approximation, considering all printed dipoles come from the same manufacturing process, and that the radiation pattern is relatively omnidirectional, which reduces the probe influence in the postprocessing steps.

### 4.2. Parameters Extraction

Once the calibration, the probe correction and the NF to FF SCT are applied to the performed measurement to obtain an accurate reconstructed FF radiation pattern, the AUT directivity, gain and complex feeding coefficients may be computed. Once again, the a priori information obtained from the Gold Antenna is used to compute such parameters. The AUT directivity can be written in terms of its PWS [15]:

(7) $$\hat{D}=\frac{{\left|\vec{P}\left(0,0\right)\right|}^{2}}{\iint_{-\infty }^{\infty }{\left|\vec{P}\left(k_{x},k_{y}\right)\right|}^{2}dk_{x}dk_{y}}$$

Assuming separability and particularizing Equation (2) in Equation (6), the directivity can be computed straightforwardly with:

(8) $$\hat{D}=\frac{{\left|P_{y}^{ind}\left(0\right)\;AF\left(0\right)\right|}^{2}}{\iint_{-\infty }^{\infty }{\left|P_{y}^{ind}\left(k_{x}\right)\;AF\left(k_{y}\right)\right|}^{2}dk_{x}dk_{y}}$$

where all the terms involved in the expression are available as previously shown.

The AUT gain is obtained using the comparison method of the planar NF techniques, which uses an auxiliary antenna of known gain. Of course, this auxiliary antenna will be the Gold Antenna. According to the theory of planar near-field measurements, the AUT gain is obtained weighting the Gold Antenna gain by a coefficient involving the measured fields of both antennas [15], and by $G_{gold}\left(0\right)$ the gain of the Gold Antenna:

(9) $$G_{aut}\left(0\right)={\left|\frac{\sum_{i}E_{aut}\left(i\right)}{\sum_{i}E_{gold}\left(i\right)}\right|}^{2}G_{s}\left(0\right)$$

where $E_{aut}$ and $E_{gold}$ are the measured NF samples for one polarization on a plane of the AUT and the Gold Antenna respectively. To avoid the use of planar NF samples that are not available from the SCT-MPMS, the summations in (9) can be related to the plane wave spectrum of both antennas:

(10) $$G_{aut}\left(0\right)={\left|\frac{P_{aut}\left(0,0\right)}{P_{gold}\left(0,0\right)}\right|}^{2}G_{s}\left(0\right)$$

where $P_{aut}$ and $P_{std}$ are the PWS of the AUT and Gold Antenna respectively, as retrieved from the measurement in the SCT-MPMS without any normalization factor. 

It is important to note that this approach will not work so well when the main beam of the radiation pattern is not fully captured within the reliable region of the multiprobe system, as in the proposed system that characterizes linear arrays with different beam-tilted radiation patterns. Because the gain measurement is based in comparing the power received by the Gold Antenna and the AUT, the power that is not captured by the probe array will be neglected. This introduces an error in the gain. Usually most of the power is concentrated in the main beam, so measurements with good accuracy can be obtained if it is covered by the reliable margin. If the antenna is steered, the angular margin may not fully capture the main beam. For example, if the main lobe is cut by the reliable angle at an amplitude level of −10 dB, this means that the measurement system is neglecting at least more than −10 dB of radiated power. This will have a considerable effect in the computation of the gain. As it will be shown in Section 5, truncation levels below −20 dB are enough to obtain acceptable gain results. For improved accuracy, larger MPMS may be implemented to extend the reliable angular margin of the measurement, but the switching matrix complexity will increase as well.

The computation of the array complex feeding coefficients provides useful information of the feeding network allowing to diagnose malfunctions on the amplitude or phase of each element. It is well known that the array factor is the Fourier Transform of the element’s complex excitations. For the case of a linear array settled in the y axis this relationship can be expressed as:

(11) $$AF\left(k_{y}\right)=\overset{M-1}{\underset{m=0}{\sum }}B\left[n\right]\;e^{-jnk_{y}D}$$

where $B\left[n\right]$ is the complex feeding coefficients and D is the separation between elements of the AUT array. Inverting Equation (11) by means of an inverse Fourier transform allows these coefficients to be extracted directly. 

It is worth mentioning that the proposed SCT-MPMS can calculate not the only the parameters that are treated within this paper, but also the -3dB beamwidth or side-lobe level (SLL) may be extracted, among others to validate antennas in batch measurements.

## 5. Measurement Results

**Figure 5:**
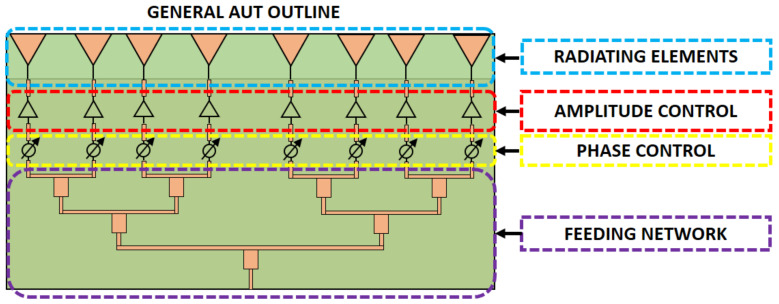
Architecture of the linear array used as AUT to validate the proposed measurement system.

In this section, the proposed SCT-MPMS is validated using the demonstrator introduced in Section 2. A linear array of 8 printed dipoles arranged in a column operating at X-band has been manufactured for this purpose. The array integrates a phase-shifter circuit to perform electronic scanning to show how the system can perform measurements at different pointing directions simultaneously. Figure 5 depicts the architecture of the AUT.

To demonstrate the capabilities of the proposed measurement system, the linear array is measured in the developed demonstrator and in the Compact Antenna Test Range (CATR) of the Technical University of Madrid (UPM). The CATR measurements will be used as a reference to assess the performance of the demonstrator. Before performing the measurements, the Calibration and Gold antenna characterization must be performed to obtain reliable measurements. For these two steps, the same antenna is used: an identical linear array of 8 elements but without beam steering capabilities in this case. This antenna is fully tested in the CATR to extract its gain and pattern characteristics. Then it is placed in a planar scanner to perform the linear measurement and finally in the demonstrator to extract the calibration coefficients of each probe. Once the system is fully initialized, it can be used to perform fast measurements of linear array AUT’s. The two selected AUT’s are measured and postprocessed.

**Figure 6:**
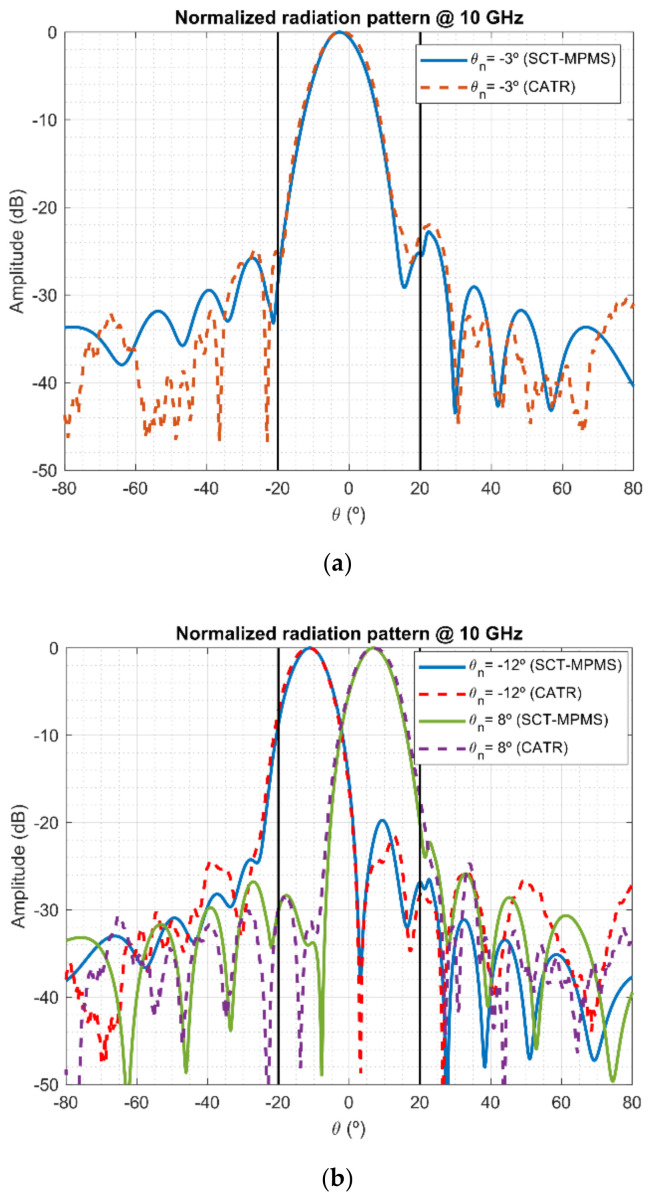
Comparison of the measurements on the SCT-MPMS demonstrator and Compact Antenna Test Range (CATR) for different pointing directions of the AUT: (**a**) near to boresight and (**b**) far from boresight.

Figure 6 shows the measurements of one of the AUTs for three different pointing directions at 10 GHz for both the demonstrator and the CATR. In addition, the reliable region $\chi_{1}$ = 20° has been depicted to stress the capabilities of the Gerchberg-Papoulis algorithm to extrapolate the radiation pattern. Even for the extreme pointing direction −12°, the postprocessing is able to reconstruct the main lobe with excellent accuracy. Outside the reliable region, the differences between the demonstrator and the CATR are more significant, but the agreement between both reaches down to pattern levels of −30 dB. This is a reasonable level of accuracy considering the fast-testing capabilities of the proposed system.

**Figure 7:**
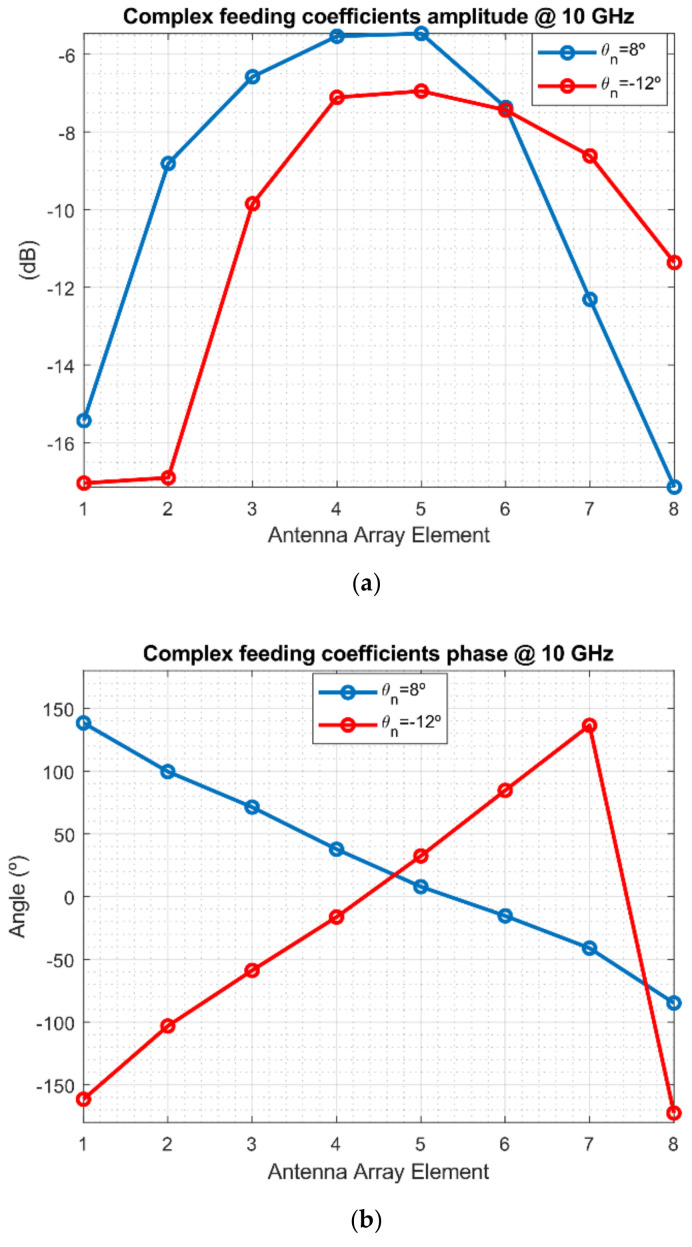
Complex feeding coefficients for the measured AUT for 2 pointing angles: (**a**) amplitude and (**b**) phase.

The retrieval of the complex feeding coefficients of the array constitutes a powerful tool to evaluate the performance of the AUT and diagnose the source of errors in the radiation pattern. The amplitude and phase of the coefficients have been extracted to assess the amplitude and phase distribution generated by the array feeding network. Figure 7 shows the amplitude and phase plots of both measured AUT coefficients for several pointing directions. In this case, the AUT has been designed to have a Taylor amplitude shape with a tapper of −10 dB. The phase shows a linear behavior with different slopes depending on the pointing direction. It is noticeable that a certain error in the amplitude is present, but this is due to imperfections in the design, limitations of the reconstruction algorithm, among other defects. Furthermore, by looking at this figure, the primary goal would be to find anomalies in the amplitude levels of each element or inappropriate phase values, hence leading to a faulty antenna element.

Finally, Table 1 shows the directivity, gain and antenna losses of the measured AUT. This serves as a comparison summary for all pointing angles. Good agreement is observed between both measurement systems in the directivity levels. Nevertheless, in the case of the losses, the demonstrator shows a significant deviation with respect to the CATR for the pointing direction −12°. The reason for this is that the demonstrator probe array is unable to cover enough angular range so the main lobe of the radiation pattern is completely acquired. It can be appreciated how the black lines delimiting the reliable region cuts the main beam at level of −10 dB. This is an excessive level of truncation which introduces an extra loss in the computed gain. For the rest of the cases, the truncation level is below −20 dB, which is enough to obtain a good accuracy in the gain computation.

## 6. Potential and Limitations of the SCT-MPMS

The concept introduced in this paper is targeted to antenna measurements in mass productions scenarios. In some radar and communication applications, throughputs in the order of thousands of antennas per year are common, so manufacturers face the challenge of testing a high number of devices in limited periods. Radiation pattern measurements are usually the bottleneck in the antenna testing process chain. For example, a planar NF measurement of an AUT like the one presented in Section 5 can take between 5 and 10 min assuming a moving probe in scan-step mode in the X-Y axis. If the considered AUT operates a several frequencies and pointing angles, the number of measurements per point in the acquisition plane multiplies. This may require a reduction in the positioner speed or even change the probe positioner to a step-step mode, which considerably increases the measurement time. In addition, the time required to properly place and align the AUT system must be considered to, which can take a few minutes too in a standard measurement system.

The proposed SCT-MPMS aims to face this limitation by reducing the measurement time to the minimum. Once the antenna is placed in the system, only the near-field samples over a line are recorded using a multiprobe system. Thus, no mechanical moving of probe or AUT is required which takes up most of the time in conventional antenna measurements. The measurement time will be the one required to switch between all probes and AUT pointing angles, which is in the order of seconds as it is purely electronical. Moreover, the proposed concept is compact, so it can be easily integrated in an ad-hoc anechoic box. If adequate mechanical fixtures are incorporated to this box in the form of alignment pins or support structures, designed specifically for the AUT footprint, the time required to mount the antenna properly can also be reduced to the order of seconds. Finally, all these tasks can be implemented with low complexity by robotic equipment without requiring intervention of a human operator, automatizing the complete measurement process.

The improved capabilities of the SCT-MPMS come with a series of limitations. Unlike a standard near-field measurement set-up where the probe is mounted on an adjustable X-Y-Z positioner, the proposed concept fixes the number of probes, their location and distance to the AUT. Thus, the system must be specifically designed for the model of the antenna to be measured. Depending on the AUT physical dimension, S_AUT,_ a different number of probes is required to cover an extension up to a given reliable angle $\chi_{1}$. As discussed in the previous sections, this angle should be selected so that it covers all the AUT radiated power down to a level of −20 dB. On the other hand, the higher the AUT-probe separation, the more probes that are required to cover a given reliable range. Therefore, the multiprobe array is placed at a distance around 5 wavelengths which is considered the closest point safe from mutual interaction between AUT and probe. If any of these parameters (frequency, antenna size, or radiation pattern) changes significantly, the proposed system should be redesigned for the new antenna model by changing the AUT-probe distance, number of probes and its spacing. The postprocessing steps must also be adapted and optimized depending on the antenna model, but this only requires software modifications. 

Measuring the NF over a single cut comes with additional accuracy costs. The proposed system relies on preliminary measurements of a Gold Antenna and it is assumed than the radiation pattern in the individual element plane is not affected by manufacturing errors. This is a reasonable assumption as most of the manufacturing errors affect the feeding network, as compared to the single element which usually has a rather simple quasi-omnidirectional pattern. This is a recurrent topic of single cut measurement techniques so one should be aware of the limitations as discussed in [8,9,10,11]. As a general guideline, the higher directivity in the array plane, the single cut measurement becomes more accurate, because the array factor dominates the individual element pattern. Therefore, the proposed system is not recommended for arrays with a low number of elements (less than 6) due to the limitations of single-cut transformation techniques. 

The truncation of the NF highly influences the accuracy of the results, as discussed in the previous section. As shown in Section 4, an excessive level of truncation leads to significant deviations in the gain calculation, and the accuracy of the retrieved pattern is degraded too. This is a source of error shared with conventional planar near-field measurements [14] and must be addressed with careful selection of the reliable angle, considering the AUT pattern and beam steering configurations. 

In summary, the implementation of the proposed SCT-MPMS concepts entails a careful design process where the specific AUT physical and electrical parameters must be analyzed to ensure an accurate and cost-effective measurement process. After an adequate system design (number of probes and spacing, probe-AUT distance, postprocessing routines configuration), the SCT-MPMS becomes an extremely efficient system with measurement times negligible as compared with traditional near-field systems.

## 7. Conclusions

In this paper, a fast, cheap and reliable measurement system for linear phased arrays has been proposed. The system relies on the power behind antenna measurement postprocessing techniques to relax the hardware requirements of the system and leave most of the complexity to the software. The operating principle consist of measuring the AUT NF with a multi-probe system in a set of discrete locations arranged in a line on the AUT array plane. By using SCT’s combined with extrapolation techniques, the FF on the same plane can be computed. This linear measurement is complemented with a previous full testing of a Gold antenna for an accurate postprocessing and to retrieve other parameters like AUT directivity, gain and array complex feeding coefficients. The proposed system becomes an extremely useful tool when a high number of nominally identical AUTs need to be measured since, after the previous calibration and initialization steps, linear arrays can be measured in seconds.

An X-band 16-probe demonstrator of the proposed system has been developed and explained in detail. All steps required to operate the demonstrator have been introduced: calibration, Gold antenna characterization, measurement and postprocessing. Finally, some measurement results have been shown for electronically steered arrays, obtaining a pretty good agreement with standard anechoic chamber measurements. Thus, the proposed multiprobe linear measurement system is a promising solution to measure linear phased arrays in a quick, cheap, and efficient manner. 

The authors propose some improvements to the actual system such as: arranging the probes in a circular sector fashion to improve the reliable region, using an orthogonal array of probes to capture information of the other principal plane improving the accuracy of the system, and using synchronized Software Defined Radio receivers to remove the need for VNAs and make the measurements even cheaper.

## Figures and Tables

**Table 1:** Directions @ 10 GHz.

Pointing (°)	Proof of Concept			Anechoic Chamber		
	Directivity (dBi)	Gain (dBi)	Losses (dB)	Directivity (dBi)	Gain (dBi)	Losses (dB)
−12	14.3	10.5	3.9	14.2	12.1	2.1
−3	14.6	12.3	2.3	14.5	12.5	2
8	14.3	11.8	2.6	14.3	12.1	2.2

## Data Availability

Not applicable.
